# Depressive symptoms in adolescence and adult educational and employment outcomes: a structured life course analysis

**DOI:** 10.1017/S0033291724001090

**Published:** 2024-05-31

**Authors:** José A. López-López, Kate Tilling, Rebecca M. Pearson, Mina S. Fazel, Elizabeth Washbrook, Yiwen Zhu, Brooke J. Smith, Erin C. Dunn, Andrew D. A. C. Smith

**Affiliations:** 1 Department of Basic Psychology and Methodology, Faculty of Psychology and Speech Therapy, University of Murcia, Murcia, Spain; 2 Centre for Academic Mental Health at the University of Bristol, Oakfield House, Bristol, UK; 3 MRC Integrative Epidemiology Unit, University of Bristol, Bristol, UK; 4 Department of Psychology, Manchester Metropolitan University, Manchester, UK; 5 Department of Psychiatry, University of Oxford, Oxford, UK; 6 School of Education, University of Bristol, Bristol, UK; 7 Department of Epidemiology, Harvard T.H. Chan School of Public Health, Boston, MA, USA; 8 Psychiatric and Neurodevelopmental Genetics Unit, Center for Genomic Medicine, Massachusetts General Hospital, Boston, MA, USA; 9 Department of Psychiatry, Harvard Medical School and the Massachusetts General Hospital, Boston, MA, USA; 10 Mathematics and Statistics Research Group, University of the West of England, Bristol, UK

**Keywords:** ALSPAC, depression, life course, NEET

## Abstract

**Background:**

Depression is a common mental health disorder that often starts during adolescence, with potentially important future consequences including ‘Not in Education, Employment or Training’ (NEET) status.

**Methods:**

We took a structured life course modeling approach to examine how depressive symptoms during adolescence might be associated with later NEET status, using a high-quality longitudinal data resource. We considered four plausible life course models: (1) an *early adolescent sensitive period model* where depressive symptoms in early adolescence are more associated with later NEET status relative to exposure at other stages; (2) a *mid adolescent sensitive period model* where depressive symptoms during the transition from compulsory education to adult life might be more deleterious regarding NEET status; (3) a *late adolescent sensitive period model*, meaning that depressive symptoms around the time when most adults have completed their education and started their careers are the most strongly associated with NEET status; and (4) an *accumulation of risk model* which highlights the importance of chronicity of symptoms.

**Results:**

Our analysis sample included participants with full information on NEET status (*N* = 3951), and the results supported the *accumulation of risk model*, showing that the odds of NEET increase by 1.015 (95% CI 1.012–1.019) for an increase of 1 unit in depression at any age between 11 and 24 years.

**Conclusions:**

Given the adverse implications of NEET status, our results emphasize the importance of supporting mental health during adolescence and early adulthood, as well as considering specific needs of young people with re-occurring depressed mood.

## Introduction

Depression is a common mental health disorder that often starts during adolescence, with potentially important future consequences for social, economic, and other areas of adult life (Fazel, Hoagwood, Stephan, & Ford, 2014; Thapar, Collishaw, Pine, & Thapar, 2012; World Health Organization, 2023). A better understanding of its potentially causal mechanisms is likely to be instrumental in the design of effective and impactful public health interventions and prevention for young people (Davey & McGorry, 2019).

A major negative outcome in adulthood that has been linked to adolescent depression in previous studies is ‘Not in Education, Employment or Training’ (NEET) status (Basta et al., 2019; Hakulinen et al., 2019; López-López et al., 2020; Veldman et al., 2021). NEET status affects over one-in-ten 16–24-year olds in England (Department for Education, 2019). Furthermore, young adults who are NEET are a high-risk group for adverse health, personal, and societal outcomes, such as societal exclusion, substance use, crime, and suicidal behaviors (Benjet et al., 2012; Fergusson, Horwood, & Woodward, 2001; Gariépy, Danna, Hawke, Henderson, & Iyer, 2021). Indeed, a previous study found that young people from the UK who showed depressive symptoms in the first years of adulthood were about 4 times more likely to be NEET at age 24 than their counterparts with no history of depressive symptoms, and the odds increased to about 6 to 1 for those who had also experienced depressive symptoms earlier on (López-López et al., 2020). However, the amount and robustness of the scientific evidence on the role of adolescent depression as a predictor of adult NEET status remains scarce. Disentangling the role of adolescent depression in subsequent NEET status is therefore a relevant and important question to address from all research, clinical, and policy perspectives.

One of the challenges faced by researchers interested in examining the association between depressive symptoms and NEET status is that data can only be obtained through observational methods, such as cross-sectional or cohort studies. Nonetheless, the availability of multi-wave longitudinal data from prospective cohort studies provides an opportunity to explore the potential mechanism underlying adolescent depressive symptoms and adulthood NEET using a life course approach, which involves analysis of the effect of the exposure to a risk factor on an outcome at a later stage in life (Kuh & Ben-Shlomo, 2004). In particular, identification of time-varying associations between depressive symptoms and NEET is made possible by recent developments of structured life course approaches, where a closed set of life course models (or hypotheses) that describe how the association of interest might work are encoded and compared within a single modeling framework (Mishra et al., 2009).

To examine how depressive symptoms during adolescence might be associated with later NEET status, we consider four plausible life course hypotheses (Ben-Shlomo, 2002; Smith, Smith, & Dunn, 2022). The first is an *early adolescent sensitive period model* implying that depressive symptoms during the first stage of adolescence have a greater impact in increasing the risk of later NEET status relative to exposure at other stages (Schaefer, Cheng, & Dunn, 2022). In the same vein, a *mid adolescent sensitive period model* could be proposed which states that depressive symptoms during the transition from compulsory education to adult life might be more deleterious regarding NEET status (Rodwell et al., 2018). A third possibility is a *late adolescent sensitive period model*, meaning that depressive symptoms around the time when most adults have completed their education and started their careers are the most strongly associated with NEET status (Benjet et al., 2012; Dunn et al., 2018). Last, an *accumulation of risk model* may also be considered (Hale & Viner, 2018; Rutter, Maughan, Mortimore, & Outston, 1979). The latter highlights the importance of chronicity of depressive symptoms, rather than specific time periods, as driving risk for NEET status in young adulthood (López-López et al., 2020). In this paper, we aimed to compare these four life course hypotheses using a highquality longitudinal data resource. We focused on the age range spanning from early adolescence to early adulthood, as most people first experience depressive symptoms during these years, and there are preventative and targeted interventions that could be supported further, and better adapted to the most optimal stages if the consequences of depressive symptoms during these stages were better understood.

## Methods

### Design and participants

Data came from the Avon Longitudinal Study of Parents and Children (ALSPAC), a prospective, longitudinal birth-cohort of children (Boyd et al., 2013; Fraser et al., 2013). Pregnant women residing in Avon, UK with expected dates of delivery 1st April 1991 to 31st December 1992 were invited to take part in the study. The initial number of pregnancies enrolled was 14 541 (for these at least one questionnaire was returned or a ‘Children in Focus’ clinic had been attended by 19/07/99). Of these initial pregnancies, there was a total of 14 676 foetuses, resulting in 14 062 live births and 13 988 children who were alive at 1 year of age.

When the oldest children were approximately 7 years of age, an attempt was made to bolster the initial sample with eligible cases who had failed to join the first phase of the study. As a result, when considering variables collected from the age of 7 onwards (and potentially abstracted from obstetric notes) there are data available for more than the 14 541 pregnancies mentioned above. The number of new pregnancies not in the initial sample (known as Phase I enrolment) that are currently represented on the built files and reflecting enrolment status at the age of 24 is 913 (456, 262 and 195 recruited during Phases II, III and IV respectively), resulting in an additional 913 children being enrolled. The phases of enrolment are described in more detail in the cohort profile paper and its update (Boyd et al., 2013; Northstone et al., 2019). The total sample size for analyses using any data collected after the age of 7 is therefore 15 454 pregnancies, resulting in 15 589 fetuses. Of these 14 901 were alive at 1 year of age (Northstone et al., 2019).

Study data were collected and managed using REDCap electronic data capture tools hosted at the University of Bristol.1 REDCap (Research Electronic Data Capture) is a secure, web-based software platform designed to support data capture for research studies (Harris et al., 2009). Please note that the study website contains details of all the data that is available through a fully searchable data dictionary and variable search tool (http://www.bristol.ac.uk/alspac/researchers/our-data/).

### Measures

#### Outcome

**NEET:** at age 24, participants were asked to complete a checklist of whether they were currently engaged in any education/employment activities. Participants were classified as NEET if they indicated they were not engaged in any of the following: full- or part-time paid work; government training scheme; full-time education; self-employment; other self-reported activity indicating a job or training (e.g. maternity leave, part-time education). Our definition is in line with that used by the UK Office for National Statistics (Department for Education, 2019).

#### Exposure

**Depressive symptoms:** the Short Mood and Feelings Questionnaire (SMFQ) is a self-reported 13-item questionnaire measuring depressive symptoms widely used for adolescent screening and monitoring purposes (Angold et al., 1995). Responders are asked to appraise each phrase as descriptive of their experiences ‘most of the time’, ‘sometimes’, or ‘not at all’ in the past two weeks. The total score ranges between 0 and 26, with higher scores indicating more depressive symptoms. ALSPAC participants completed this questionnaire on nine occasions between the ages of approximately 11 and 24 years.

#### Covariates

**Sex:** child biological sex at birth was used in this study.

**Intelligence Quotient:** given its potential association with the outcome of this study (Gladwell, Popli, & Tsuchiya, 2022), child IQ was measured at the eight-year clinic visit using the Weschler Intelligence Scale for Children (Weschler, Golombok, & Rust, 1992), a well-validated instrument that provides scores of verbal and non-verbal IQ, which we averaged for the analyses.

**Maternal age:** the age of each mother (in years) when they gave birth to the study participants was also used.

**Maternal post-natal depression:** eight weeks after giving birth, mothers completed the Edinburgh Post-Natal Depression Scale (Cox, Holden, & Sagovsky, 1987), a 10-item instrument intended to measure postpartum depression among pregnant women and new mothers. Total scores range between 0 and 30, with higher scores suggesting more severe symptoms. Maternal depression is associated with our exposure (offspring depression at all ages) both via genetic and environmental mechanisms, and our outcome NEET where having a depressed parent may also directly influence opportunities/access to education and work.

**Maternal education:** at the child’s eight-year clinic visit, mothers were asked whether they had finished compulsory school (i.e. at least age 16 qualifications v. no qualifications).

**Socioeconomic status (SES):** we used the earliest available information (usually from pregnancy) about mother’s and partner’s occupation to define a binary variable capturing low SES (unskilled, partly skilled, and skilled manual workers) and high SES (professional, managerial, technical, and skilled non-manual workers) (Galobardes, Shaw, Lawlor, Lynch, & Davey Smith, 2006). We took the highest level of either parent to define SES at the family level.

**Specific learning difficulties (SpLD):** we included maternal report at age 9 on diagnosis of specific learning difficulties (SpLD, dyslexia, dyspraxia, dysgraphia, dysortografia, and dyscalculia), and retrospective self-report at age 22 on receiving support for any of these at school, university, or work. This covariate was only included in sensitivity analyses.

**Neurodiversity:** we included maternal report at age 9 on diagnosis of Autism Spectrum Disorders (ASD), and retrospective self-report at age 22 on receiving support for any of these at school, university, or work. This covariate was only included in sensitivity analyses.

### Analysis

After conducting univariate and bivariate analyses to explore the distribution of the exposure and the covariates, we examined the time-dependent associations between adolescent depressive symptoms and NEET status at age 24 using a structured life course modeling approach (SLCMA). This analysis framework enabled us to simultaneously examine an *early sensitive period model* (using three SMFQ measures between 11 and 14 years), a *mid sensitive period model* (comprising three SMFQ measures between 17 and 19 years), a *late sensitive period model* (including three SMFQ measures at 22–24 years), and an *accumulation of risk model* (where all nine SMFQ measures were considered). Each of the models were previously adjusted for the list of covariates presented in a previous section. We conducted our analysis within a SLCMA framework with continuous exposure variables as proposed by Smith et al. (2016).

The SLCMA compares the theoretical models using a two-stage strategy. In the first stage, the SLCMA uses variable selection of the key variables from our 4 life course models (S1, S2, S3 and A; see Table 1) to identify the most likely theoretical model, or combination of models, given the observed data. Users of the SLCMA typically apply least angle regression (LARS) for variable selection and an accompanying elbow plot of *R*^2^ values to aid in selection of the best-fitting hypotheses (Smith et al., 2015). As our outcome variable was binary, we used a series of least absolute shrinkage and selection operator (LASSO) estimates instead of LARS, and an elbow plot of the McFadden’s pseudo-R index (McFadden, 1973). The second stage consists of estimating parameters and confidence intervals for the models previously selected. Typically, the second stage employs post-selection inference methods to overcome the bias in hypothesis testing and confidence intervals that is introduced by variable selection in the first stage. In both stages, all proposed and selected models are adjusted for covariates. Although the SLCMA considers all the models described in Table 1, and their potential combinations, it does not require presentation or interpretation of all the models, only the model that is identified as the most likely given the observed data.

### Missing values

Due to attrition, there were missing data for exposures and covariates. Exclusion of participants with missing data may result in unrepresentative samples and consequently selection bias, hence we used multiple imputation by chained equations (MICE) (Van Buuren, 2007). Our analysis sample included participants with full information on the outcome (NEET status), with MICE used to impute missing data for the exposure variables as well as covariates. We used academic results at the end of compulsory education as an auxiliary variable in the imputation models, which also contained all non-missing values of exposures, covariates, and outcome. Thus, the imputation model was compatible with the SLCMA as it contained all variables that would be considered in the SLCMA. This process produced 20 imputed data sets. We used stacked imputation (Wood, White, & Royston, 2008) for the first stage of the SLCMA to identify the most likely theoretical model in an averaged-across-imputations dataset. Briefly, stacked imputation involves ‘stacking’ the 20 imputed datasets on top of each other to create a single dataset with the average covariance structure of the imputed datasets. LASSO estimation is then employed on the stacked dataset, with each row given a weight of 1/20 to preserve the analysis sample size. In the second stage, we employed bootstrap methods on the stacked dataset to calculate *p*-values and 95% confidence intervals for the parameters estimated by the LASSO models, overcoming the bias introduced in the first stage.

The analyses were conducted in the R statistical environment (R Core Team, 2021) using packages lars (Hastie & Efron, 2013), glmnet (Friedman, Hastie, & Tibshirani, 2010), mice (van Buuren & Groothuis-Oudshoorn, 2011), and boot (Canty & Ripley, 2021).

## Results

### Descriptive results

Our analysis sample included participants with full information on NEET status (*N* = 3951). Of these, 2582 (65.4%) were female and 359 (9.09%) were considered to have NEET status. The median age on completion of the checklist used to inform NEET status was 23 years 10 months (minimum 22 years 11 months, maximum 24 years 11 months). The mean SMFQ scores before imputation ranged between 3.8 at age 11 and 7 at age 24. The average maternal age was 29.4 years, with 2707 (86.4%) achieving at least age 16 qualifications (Table 2).

### SLCMA results

**Figure 1:**
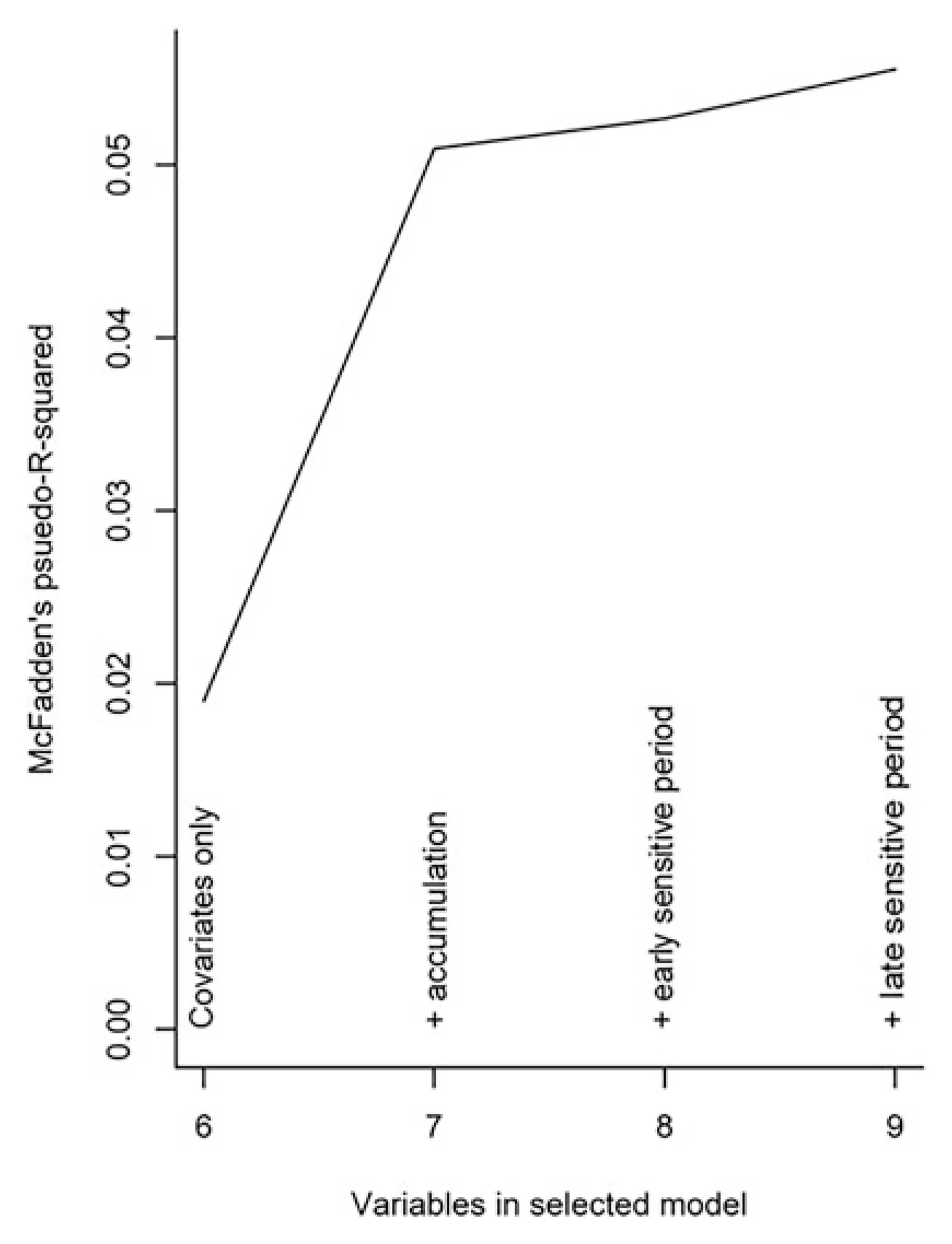
Elbow plot illustrating the variable selection process. The simplest model contains only covariates. The LASSO procedure identifies the single key variable showing the strongest association with the outcome (in this case the key variable representing the accumulation of risk model), then the combination of two key variables with the strongest association, and so on. The selected model is indicated by a sharp concave bend (the ‘elbow’) at which adding more variables does not substantially increase pseudo-*R*^2^.

The SLCMA identified the *accumulation of risk model* as the most supported by the observed data. Examination of the elbow plot (Fig. 1) showed there was no substantial improvement in model fit associated with adding further variables to the model.

The fitted accumulation of risk model estimated that the odds of NEET increase by 1.015 (95% CI 1.012–1.019) for an increase of 1 unit in accumulated SMFQ score at any age between 11 and 24 years. Alternatively, an increase of 12 units (corresponding to a change from no symptoms to severe symptoms) at any age implies an increase of 1.202 (95% CI 1.158–1.249) in the odds of NEET. The bootstrap *p*-value of this estimate was *p* < 0.00005 in the stacked multiply-imputed data and in every one of the 20 imputed datasets.

Table 3 shows how the odds of NEET increase for illustrative values of accumulated SMFQ score. For example, experiencing severe symptoms (defined by an SMFQ score of 12 or more) on one measured occasion, compared with a median SMFQ score, is associated with an increase in the odds of NEET of at least 1.240 (95% CI 1.187–1.296). Alternatively, experiencing severe symptoms on all occasions, compared with a median SMFQ score, is associated with an increase in the odds of NEET of at least 2.629 (95% CI 2.162–3.210). Given that the event of interest can be considered as rare (less than 10% of the sample participants were classed as NEETs at 24 years), our results can also be interpreted in terms of relative risks.

### Sensitivity analyses

As the final measurement of the exposure variable was at age 24 years, and the outcome was measured at age 24 years, there could be an argument for reverse causality. We ran a sensitivity analysis removing the age 24 exposure measurement from the analysis (thus the *accumulation of risk model* and *late sensitive period model* contained only exposures up to age 23 years). The accumulation model was still the most supported by the observed data, and the odds ratio for an increase of one unit in accumulated SMFQ score was 1.017, hence there were no changes to the conclusions of our analyses.

With regards to specific learning difficulties and neurodiversity, the age 9 measures could be considered as potential confounders as they precede the measurement of the exposures (first measurement is age 11), whereas the age 22 measures could be considered as time-varying covariates as they relate to the period during which the exposures were measured. We repeated our analysis, including these four measures as additional covariates. The accumulation model was still the most supported by the observed data, and the odds ratio for an increase of one unit in accumulated SMFQ score was 1.015, hence there were no changes to the conclusions of our analyses.

## Discussion

To the best of our knowledge, this is the first study examining the association between adolescent depressive symptoms and NEET status in adulthood from a SLCMA perspective. Using a large UK sample, we tested different life course hypotheses and our results support the accumulation of risk model in preference to the early sensitive period, the mid sensitive period, and the late sensitive period models.

The association examined in this paper has been the focus of previous research in this field, with several studies linking adolescent depression to later unemployment when examining the general population (Clayborne, Varin, & Colman, 2019; Fletcher, 2013). Conversely, a study using participants seeking help from mental health services (rather than general population) found no association between depressive symptoms in adolescence and NEET in adulthood (O’Dea et al., 2016).

The interpretation of our findings suggests that depressive symptoms during adolescence and early adulthood may interfere with achievement of the milestones that enable access to the job market or to higher education. Some of the core symptoms of depression include lack of motivation, low energy, and feelings of hopelessness regarding the future. These cognitions could potentially impact the developing adolescent’s approach to their studies, future job choices and feelings about themselves – including their self-confidence and feelings of general capacity. A potential mediator of the association observed in the present study is academic results. In this vein, a previous study using the ALSPAC sample found evidence of bi-directional associations between depressive symptoms and educational attainment between 11 and 18 years (López-López et al., 2021). Further potential pathways include substance use, which has been found to be associated with NEET status before (Fergusson et al., 2001; Gariépy et al., 2021); relationship breakdowns – including social isolation and social anxiety and withdrawal – and school refusal, among others.

With regard to implications for research and clinical practice, our results suggest that the longer a person is depressed during adolescence, the higher the risk of being NEET in adulthood. According to this, the key point from a public health perspective is not to identify when depression first occurs, but rather to treat it as soon as it is detected in young people and to consider specific needs of young people with re-occurring depressed mood (both in terms of alternative mental health interventions and of the impact depression might have in other key areas of adolescent and early adult life). This trend also suggests that showing depressive symptoms at one particular time point does not necessarily worsen the prospects for young people in their transition to adulthood over another time. On the other hand, those who have longer lasting severe symptoms could be considered at high risk and be prioritized for access to mental health services.

It is also possible that those with chronic symptoms have further comorbidities which make accessing mental health support as well as education more difficult. Among these potential comorbidities are specific learning difficulties and neurodiversity. As described at the end of the results section, a sensitivity analysis including these provided further support for the accumulation model over the other life course hypotheses. This suggests that specific learning difficulties and/or neurodiversity are unlikely to be confounding variables, but they may have a role as an effect moderator (e.g. exacerbating the influence of depression). In particular, autistic traits may increase exposure to bullying, risk behavior, and limit access to social support, which could in turn exacerbate the effect of depression on educational and employment outcomes (Ly, Heron, Rai, & Wright, 2023; Rai et al., 2018). There are well-known disparities in access to mental health services. Person-centered support for those young people with both depression and educational difficulties early on may prevent NEET. Current services may not be sufficiently accessible for those with chronic depression, and it is important to understand why.

Strengths of this study include use of a large, high-quality data resource. The availability of multiple time points for the exposure allows for defining and empirically examining several hypotheses that go beyond single time point association or an ever v. never exposed comparison. To the best of our knowledge, this is the first analysis to employ a structured approach to examine the association between depressive symptoms in adolescence and NEET. By adopting a structured approach (the SLCMA), we restricted our analyses to pre-specified hypotheses, rather than developing a hypothesis based on our observations.

With regards to limitations, this is an observational study and therefore the associations that we found are prone to confounding. We tackled this by adjusting for a set of baseline covariates commonly discussed in the literature (see methods section), but they do not encompass all potential sources of unobserved heterogeneity. We also found large amounts of missing data in some of the analyzed variables, which we addressed using MICE. Furthermore, the set of life course hypotheses tested in this study is not necessarily an exhaustive list, meaning other models might be proposed and tested in the future to improve understanding of this association. Last, the generalizability of the findings is yet to be determined and future work exploring other longitudinal cohorts with repeated assessments of depressive symptoms and educational and employment outcomes – such as the 1958 National Child Development Study (NCDS58), the 1970 British Cohort Study (NCS70), and the Millennium Cohort Study (Millenium) – could help validate our findings.

In conclusion, this study provides some evidence that experiencing depressive symptoms throughout adolescence and early adulthood may have a cumulative effect as a risk factor for becoming NEET at the start of adult life. Given the adverse implications of NEET status, our results emphasize the importance of providing mental health support integrated with other agencies during adolescence and early adulthood. Our results also indicate that chronicity plays a role, meaning the risks for children and adolescents who are chronically depressed (e.g. those with limited resources since childhood and/or do not respond to current treatment) are amplified. This also highlights the importance of monitoring depressive symptom trajectories in children and adolescents in clinical settings, rather than taking each clinical assessment as a standalone diagnostic data point.

## Figures and Tables

**Table 1:** Life course models investigated for the relationship between depressive symptoms in adolescence and later NEET status

Model	Description	Formula	Key variables
Covariates only	NEET status does not depend on depressive symptoms score at any of the ages 11 to 24.	logit(*Y*) = *b*_0_ + *b*_1_(covariates)	None
Early sensitive period	NEET status depends on the average depressive symptoms score at ages 11 to 14.	logit(*Y*) = *b*_0_ + *b*_1_ *S*_1_ + *b*_2_(covariates)	*S*_1_ = *X*_11_ + *X*_13_ + *X*_14_
Mid sensitive period	NEET status depends on the average depressive symptoms score measured at ages 17 to 19.	logit(*Y*) = *b*_0_ + *b*_1_ *S*_2_ + *b*_2_(covariates)	*S*_2_ = *X*_17_ + *X*_18_ + *X*_19_
Late sensitive period	NEET status depends on the average depressive symptoms score measured at ages 22 to 24.	logit(*Y*) = *b*_0_ + *b*_1_ *S*_3_ + *b*_2_(covariates)	*S*_3_ = *X*_22_ + *X*_23_ + *X*_24_
Accumulation of risk	NEET status depends on the accumulated depressive symptoms score at all ages	logit(*Y*) = *b*_0_ + *b_1_* *A* + *b*_2_(covariates)	A= *X*_11_ + *X*_13_ + *X*_14_ + *X*_17_ + *X*_18_ + *X*_19_ + *X*_22_ + *X*_23_ + *X*_24_

*Y*: NEET status at age 24. logit(*Y*) = log(*Y*/(1 − *Y*)) is the logit transformation used to link a binary outcome variable to a continuous predictor in logistic regression. log() denotes the natural logarithm. *X*_*j*_: Depressive symptoms score at age *j*.

**Table 2:** Characteristics of the analysis sample (*N* = 3951) before imputation

Outcome	Number	(%)	Missing	(%)
Status at age 24			0	(0.0%)
In education	450	(11.4%)		
In full-time employment^a^	2575	(65.2%)		
In other employment	476	(12.0%)		
In training^b^	91	(2.3%)		
NEET	359	(9.1%)		
Exposure	Mean	(S.D.)	Missing	(%)
Depressive symptoms at age 11	3.8	(3.38)	784	(19.8%)
Depressive symptoms at age 13	4.0	(3.82)	888	(22.5%)
Depressive symptoms at age 14	4.9	(4.49)	1035	(26.2%)
Depressive symptoms at age 17	6.0	(5.57)	1042	(26.4%)
Depressive symptoms at age 18	6.4	(5.16)	1344	(34.0%)
Depressive symptoms at age 19	6.9	(5.92)	1605	(40.6%)
Depressive symptoms at age 22	5.6	(5.58)	1445	(36.6%)
Depressive symptoms at age 23	6.2	(5.57)	937	(23.7%)
Depressive symptoms at age 24	7.0	(6.06)	77	(1.9%)
Covariates	Number	(%)	Missing	(%)
Sex Male Female	1369 2582	(34.6%) (65.4%)	0	(0.0%)
Maternal education At least age 16 qualifications^c^ No qualifications	2707 425	(86.4%) (13.6%)	819	(20.7%)
Socioeconomic status^d^ Non-manual occupation Manual occupation	3241 495	(86.8%) (13.2%)	215	(5.4%)
	Mean	(S.D.)	Missing	(%)
IQ at age 8	106.4	(14.47)	862	(21.8%)
Maternal age (years)	29.4	(4.51)	395	(10.0%)
Maternal ante-natal depression^e^	4.1	(2.96)	523	(13.2%)
Auxiliary	Mean	(s.d.)	Missing	(%)
GCSE results^f^	5.7	(2.72)	15	(0.4%)

a Defined as 30 or more hours a week. Of these, 294 (11.4%) earned less than £1000 per month, 1286 (50.0%) earned between £1000 and £1499 per month, and 926 (36.0%) earned at least £1500 per month (69 (2.7%) missing).

b Modern apprenticeship or other government scheme, internship, teaching, or nursing training.

c Defined as having at least grade C in O level, CSE, or GCSE qualification.

d Defined as the higher of the mother’s or partner’s (if available) occupational social class.

e Depression subscale of the Crown Crisp Experiential Index, completed at approximately 18 weeks gestation.

f Capped point score, the total score of an individual’s top eight GCSE or equivalent qualifications at approximately 16 years. Higher scores indicating higher attainment.

**Table 3:** Odds ratios for NEET status at age 24 for selected values of accumulated SMFQ score at ages 11–24.

	Average accumulated SMFQ score^a^	Odds ratio^b^	(95% confidence interval)
No symptoms on any occasion	0.00	0.501	(0.435, 0.577)
Lower quartile of accumulated SMFQ score	3.11	0.770	(0.730, 0.812)
Median accumulated SMFQ score	5.00	1	
Severe symptoms on any one occasion, median score at all other ages	5.78	1.113	(1.089, 1.138)
Severe symptoms on any two occasions, median score at all other ages	6.56	1.240	(1.187, 1.296)
Upper quartile of accumulated SMFQ score	7.67	1.445	(1.341, 1.559)
Severe symptoms on all occasions	12.00	2.629	(2.162, 3.210)

a Equal to accumulated SMFQ score divided by the number of measurement occasions. Maximum observed = 22.89.

b Odds ratio (and confidence interval) is adjusted for sex, maternal education, maternal age, maternal ante-natal depression, socioeconomic status at birth, and IQ at age 8.
